# Lifestyle-related risk factors correlated with mental health problems: A longitudinal observational study among 686 male college students in Chongqing, China

**DOI:** 10.3389/fpubh.2022.1040410

**Published:** 2022-11-18

**Authors:** Bin-Wei Yang, Peng Zou, Qing Chen, Lei Sun, Xi Ling, Huan Yang, Ni-Ya Zhou, Li-Hong Wang, Lin-Ping Huang, Jin-Yi Liu, Hui-Fang Yang, Jia Cao, Lin Ao

**Affiliations:** ^1^Key Laboratory of Environmental Factors and Chronic Disease Control, School of Public Health and Management, Ningxia Medical University, Yinchuan, China; ^2^College of Preventive Medicine, Institute of Toxicology, Third Military Medical University, Chongqing, China; ^3^Department of Scientific Research and Education, Women and Children's Hospital of Chongqing Medical University, Chongqing, China; ^4^West China School of Public Health, Sichuan University, Chengdu, China

**Keywords:** lifestyle factors, mental health problems, DASS-21, college students, longitudinal observational study

## Abstract

**Aim:**

Public concerns over the mental health problems of college students are rising. Previous research show that female tend to suffer more from mental health problems than males, with few studies focusing on males. This study sought to explore the association of lifestyle-related risk factors with the prevalence of mental health problems among male college students in China.

**Methods:**

The lifestyle information and mental health status of 686 male college students from Chongqing, China, were assessed in 2014, and 582 of them were followed up a year later. Participants completed a questionnaire assessing demographic and lifestyle factors which include sleep quality, computer usage, sedentariness, physical activity, smoking, current alcohol, coke, coffee, and milk tea drinking, and current tea/fried food/baked food consumption. Mental health problems were measured using the Depression Anxiety Stress Scale-21 (DASS-21).

**Results:**

Univariate analyses indicated that age, sleep latency, sleep duration, computer usage time, milk tea drinking, and fried food consumption were potential risk factors for mental health problems (*p*'s < 0.05). Multivariate analysis further revealed that, either at baseline or during follow-up, participants with (i) more computer usage time were at a higher risk of having depression symptoms (*p*'s < 0.05) and (ii) a higher frequency of fried food consumption were associated with a higher risk of having depression, anxiety, and stress symptoms (*p*'s < 0.05). Additionally, the cross-lagged analysis showed that (i) computer usage time in 2014 is positively correlated with depression status (β = 0.106, *p* < 0.05) but not anxiety (β = 0.047, *p* > 0.05) and stress (β = 0.019, *p* > 0.05) status a year later and (ii) fried food consumption in 2014 is positively correlated with depression (β = 0.129, *p* < 0.01), anxiety (β = 0.168, *p* < 0.001), and stress (β = 0.113, *p* < 0.01) status a year later.

**Conclusions:**

Computer usage time and fried food consumption were lifestyle-related risk factors for mental health problems in male college students in Chongqing, China. These results might emphasize further preventive strategies for mental health problems, especially in male college students.

## Introduction

The World Health Organization (WHO) describes youth's mental health as a state of well-being that allows them to learn and acquire education, develop a positive sense of identity, manage their thoughts and emotions, lead a fulfilling social life, and fully participate in society (1). Accordingly, mental health researchers are becoming increasingly aware that the absence of mental illness does not necessarily translate to psychological well-being. College students face both psychological and physical challenges and adaptations associated with the transition from high school to university campus life (2). Thus, particularly young adults, such as college students, are more likely to develop poor mental health. The proportion of college students with a diagnosed mental health condition increased from 21.9% in 2007 to 35.5% in 2016–2017 on U.S. campuses over the past 10 years (3). There is limited trend data examining the prevalence of mental health problems in Chinese college students, but existing research suggests that mental health problems have become a serious health burden for Chinese college students. For example, a recent study by Cheng et al. reported that the prevalence rate of depressive symptoms among college students was 31.2%, which was higher than general population in China (4). The mental health of college students deserves public attention.

Increasing evidence indicates that in addition to trait markers, general living conditions and major life events, simple everyday behaviors that individuals can alter also affect mental health (5). Several modifiable risk and protective factors have been identified in previous studies including sleep quality (6), circadian rhythms (7), physical activity (8), body mass index (BMI) (9), and smoking (10). Besides these factors, eating and drinking habits are also reported to be associated with mental health problems. For example, a review mentioned that, eating healthy food and avoidance of alcohol have been recognized to play an important role in positively modifying medical and psychiatric diseases and their associated morbidity and mortality (11). Those eating and drinking habit factors which are common among college students, such as alcohol drinking, coffee drinking, coke drinking, fried food consumption, and baked food consumption, deserved further study. Another issue should researchers pay attention to is that Chinese people have a long history of drinking tea. A study reported that green tea amino acid and L-theanine (L-THE) were associated with several health benefits, including improvements in mood, cognition and a reduction of stress and anxiety-like symptoms (12). Based on these results, exploring the role of tea or tea products consumption in mental health among Chinese populations might have essential research value. Furthermore, young adults should be increasingly aware of the importance of a healthy lifestyle because habits are difficult to modify in adulthood (13). In this context, identifying risk and protective lifestyle factors that promote mental health is crucial for reducing the burden of mental disorders, especially in college students.

Although previous studies have identified numerous lifestyle-related risk factors associated with mental health problems in different populations, two limitations deserve researchers' attention. One limitation concerns gender differences in the prevalence of mental health problems (14) and lifestyle characteristics (15). It may be meaningful and necessary to examine the association between lifestyle-related risk factors and mental health problems in males and females separately. Another limitation is that most previous studies were cross-sectional with relatively small sample size and limited capacity to identify cause and effect relationships.

Subjects in this study were draw from the Male Reproductive Health in Chongqing College Students (MARHCS) cohort study, in which all subjects were male college students (16). MARHCS cohort study provided us a suitable research population to address the two shortcomings of previous studies mentioned above. Therefore, this study aimed to (i) identify lifestyle-related risk factors that correlate with mental health problems at baseline and follow-up respectively and (ii) explore whether lifestyle-related risk factors at baseline predict the prevalence of mental health a year later.

## Methods

### Subjects and procedures

Subjects in this study were from the MARHCS cohort study which was designed to assess the role of environmental and socio-psycho-behavioral factors on male reproduction (16). The inclusion criteria of the MARHCS cohort study includes: (i) >18 years old, (ii) 2–7 days of abstinent, and (iii) second-grade male college students. Using propaganda poster in university campuses and online recruitment, a total of 872 volunteers were recruited in June 2013 in Chongqing, China. Seventy-six subjects were excluded for the following issues: (i) ineligible abstinent duration or refusing of coming over again (thirty-three subjects), (ii) urogenital disease (thirty-six subjects), and (iii) failure of providing semen samples (seven subjects). We also recorded psychotropic drug use in the previous 3 months and found none of the subjects reported taking a psychotropic drug. Finally, the MARHCS cohort study was initiated with 796 male college students from Chongqing, China. Two follow-up studies were carried out in 2014 (*n* = 686) and 2015 (*n* = 582), respectively. The current study used the data of the MARHCS cohort study in 2014 and 2015 because we did not measure symptoms of depression, anxiety, and stress in 2013. No significant difference of mental health status, demographic, and lifestyle information was found between the followed-up students and the lost ones. Written informed consent was obtained from all subjects. Based on the results of previous studies reporting that the prevalence of depression, anxiety, and stress symptoms in Chinese college students came at 11.7% (17), 16.3% (18), and 44.6% (19), respectively, the following formula was used to calculate the sample size of the present study.


$$\begin{array}{l} N=\;\text{p}\left(1-\text{p}\right){\left(U_{1-\text{α}/2}/\delta \right)}^{2}. \end{array}$$

Finally, we calculated the sample sizes to be 159, 210, and 380, respectively. Thus, a sample size of *N* = 380 is sufficient to estimate the prevalence of depression, anxiety, and stress symptoms with an accuracy of ± 5% using a 95% confidence interval. Each volunteer received a remuneration of $30 after completing the questionnaires.

### Ethical approval

The project proposal was approved by the Institutional Review Board of the Third Military Medical University, Chongqing, China.

### Mental health problems

Mental health problems were measured using the Chinese version of the Depression Anxiety Stress Scale-21 (DASS-21), which had good reliability and validity in the Chinese population (20). DASS-21 includes three self-administered subscales (seven questions each) measuring the symptoms of depression, anxiety, and stress using 21 questions. The questions assess participants' feelings over the past week on a four-point Likert scale (0 = “not at all,” 1 = “some of the time” = 1, 2 = “a good portion of the time,” and 3 = “most of the time”). The score of each subscale is summed up and then multiplied by two according to the guidelines of DASS-21. Subscales' scores above 9, 7, and 14 indicate having depression, anxiety, and stress symptoms, respectively. The Cronbach's alpha coefficients for the DASS-21 in this study were 0.87 and 0.84 in 2014 and 2015, respectively.

### Demographic and lifestyle information

Age and race were collected *via* self-report. A single corrected instrument measured weight (kg) and height (cm). Body mass index (BMI) was calculated using the following formula: *BMI (kg/m*^2^*)* = *weight (kg) / height*^2^ *(meter)*. Body fat (%) was measured using a hand-held body fat caliper (INBODYH20B, InBody, Korea).

This study included thirteen lifestyle-related questions assessing lifestyle practices: sleep latency, sleep duration, computer usage time, time remaining seated, physical activity, current tobacco smoking, current alcohol drinking, current tea consumption, current coke drinking, current coffee drinking, current milk tea drinking, fried food consumption, and baked food consumption (11, 12, 21, 22). Sleep latency and sleep duration were assessed using questions, “How many minutes did it take you to fall asleep after going to bed in the last month?” and “What was your actual sleep time per night in the last month?” The questions that assessed computer usage time was “How much time do you spend using the computer (including both desktop computers and laptops) every day?” “Physical activity” was measured by a question as “How many times per week do you engage in physical exercise?” The answer includes “ < 1 time/week,” “1~2 times/week,” “3–6 times/week,” and “≥ 7 times/week.” “Time remaining seated” was measured by a question as the following “How much time do you remain seated every day?” The answer includes “ < 1 h/day,” “1–2 h/day,” “3–5 h/day,” “6–10 h/day,” and “> 10 h/day.” Smoking habit was assessed with the question, “Are you a never smoker, ex-smoker, or current smoker?” Drinking habits were measured with the question, “Do you drink alcohol/ tea/ coke, coffee, or milk tea?” Fried food consumption frequency was measured by a question as “How many times do you eat fried food each week?” The answer includes “ < 1 time/week,” “1~2 times/week,” “3–6 times/week,” and “≥ 7 times/week.” Baked food consumption frequency were determined using the question, “How many times do you eat baked food each week?” The answer includes “ < 1 time/week,” “1~2 times/week,” “3–6 times/week,” and “≥ 7 times/week.”

### Data analysis

**Figure 1:**
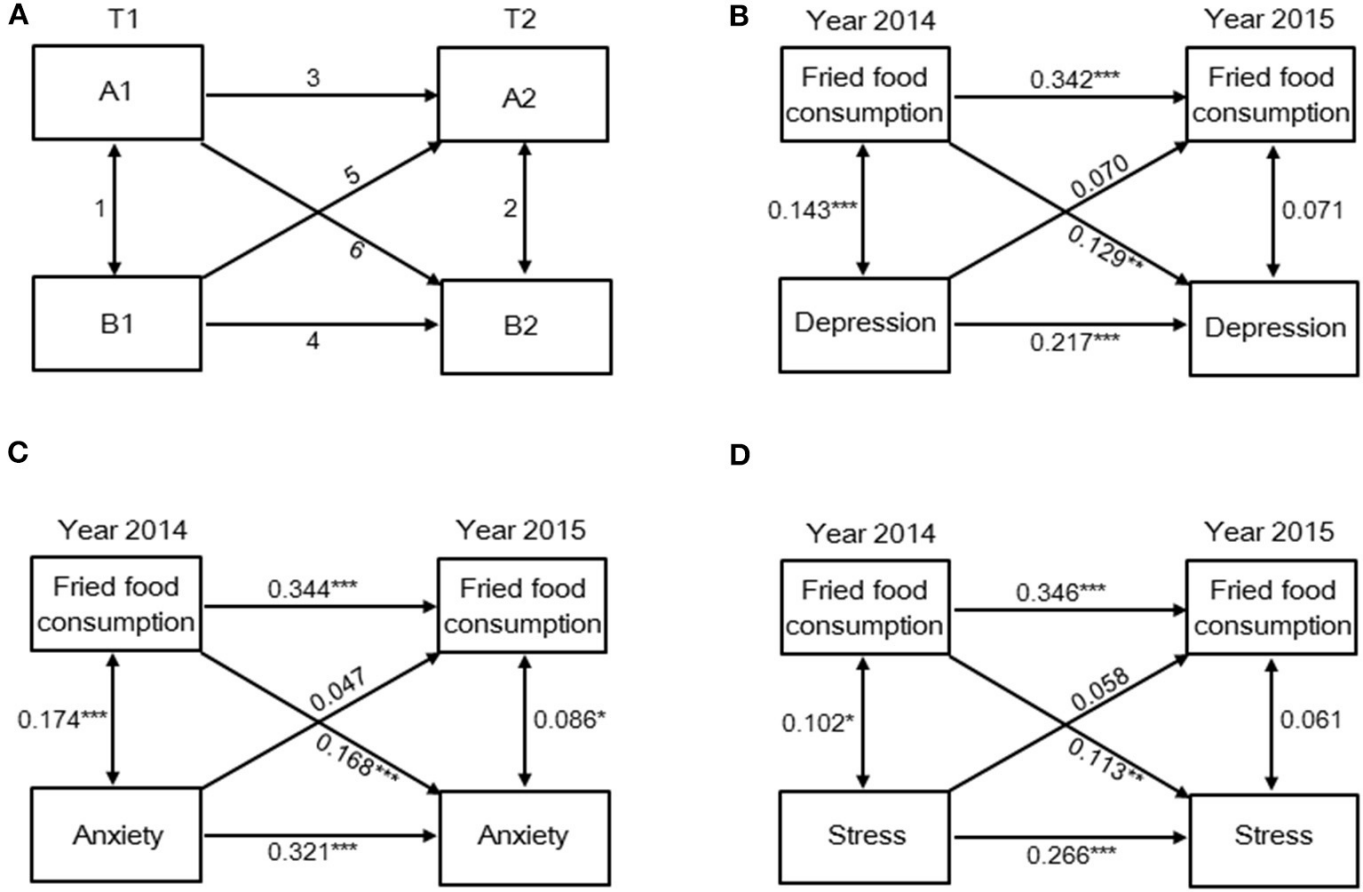
Fried food consumption is a predictor of depression, anxiety, and stress status a year later. **(A)** Cross-lagged correlational model describing three types of relationships: 1, 2 = synchronous correlations; 3, 4 = autocorrelations; 5, 6 = cross-lagged correlations. Prospective associations between fried food consumption and status of **(B)** depression, **(C)** anxiety, and **(D)** stress on the DASS-21. *p < 0.05, **p < 0.01, and ***p < 0.001.

**Figure 2:**
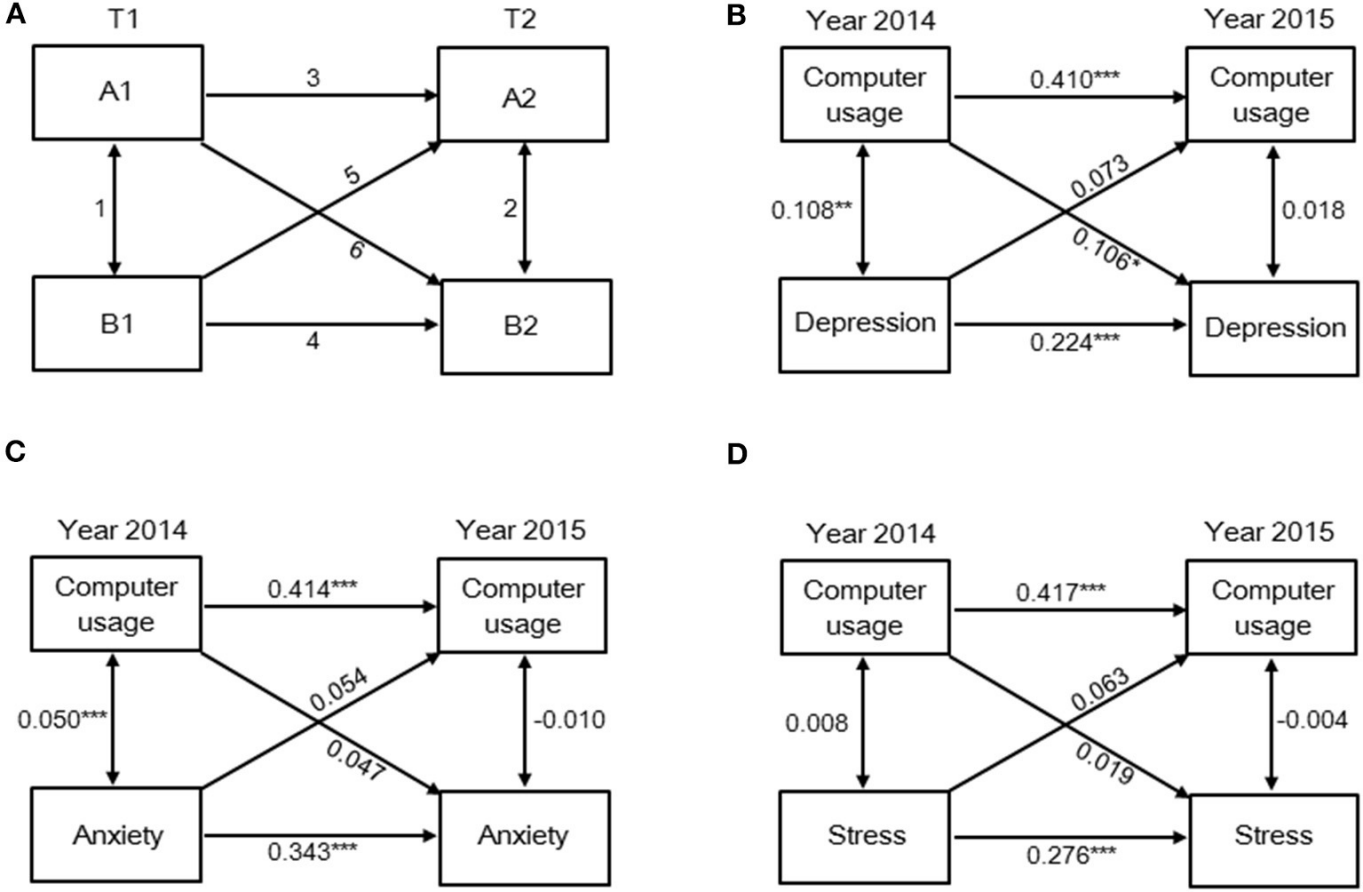
Computer usage time is a predictor of depression status but not anxiety and stress status a year later. **(A)** Cross-lagged correlational model describing three types of relationships: 1, 2 = synchronous correlations; 3, 4 = autocorrelations; 5, 6 = cross-lagged correlations. Prospective associations between computer usage time and status of **(B)** depression, **(C)** anxiety, and **(D)** stress on the DASS-21. *p < 0.05, **p < 0.01, and ***p < 0.001.

Differences in depression, anxiety, and stress symptoms were analyzed using Mann-Whitney U tests (continuous variables) and χ^2^-tests (categorical variables). Multiple logistic regressions were performed to further confirm the associations between lifestyle-related risk factors identified in the univariate analyses and prevalence of depression, anxiety, and stress symptoms. Continuous variables (age, computer usage time, sleep latency, and sleep duration) were converted into dichotomous variables according to its median values before performing logistic regressions. The prospective associations between the two lifestyle-related risk factors (computer usage time and fried food consumption) and mental health problems were examined by the cross-lagged analysis using structural equation modeling according to the methods described in previous studies (6, 23). Figures 1A, 2A describe a cross-lagged model in which variables A and B are measured at two time points (T1 and T2). The cross-lagged model include three types of relationships: (i) synchronous correlations (1 and 2), (ii) autocorrelations (3 and 4) and (iii) cross-lagged correlations (5 and 6). The lavaan package of R version 3.6.3 was used for cross-lagged analysis. IBM SPSS 22.0 Software (IBM Corp., Armonk, NY) was used to analyze the data, and statistical significance was set at *p* < 0.05, *p* < 0.01 and *p* < 0.001.

## Results

### Sample characteristics

At baseline, the median age of 686 male college students (Table 1) was 21 years. The prevalence rates of depression, anxiety, and stress symptoms at baseline were 9.62% (*n* = 66), 18.95% (*n* = 130), and 8.02% (*n* = 55), respectively, and the prevalence rates of depression, anxiety, and stress symptoms at follow-up were 6.70% (*n* = 39), 12.71% (*n* = 74), and 6.70% (*n* = 39), respectively. Demographic characteristics and lifestyle information across 2 years are detailed in Table 1 (baseline) and Table 2 (follow-up).

**Table 1:** The distribution of demographic characteristics and lifestyle factors according to the status of depression, anxiety, and stress symptoms among 686 male college students at baseline (year 2014).

**Variables**	**All subjects**		**Depression symptom**		***p*-values^a^**	**Anxiety symptom**		***p*-values^a^**	**Stress symptom**		***p*-values^a^**
		**(*N* = 686)**	**No (*n* = 620)**	**Yes (*n* = 66)**		**No (*n* = 556)**	**Yes (*n* = 130)**		**No (*n* = 631)**	**Yes (*n* = 55)**	
	**n**	**Median (25th, 75th percentile) or n (%)**	**Median (25th, 75th percentile) or n (%)**	**Median (25th, 75th percentile) or n (%)**		**Median (25th, 75th percentile) or n (%)**	**Median (25th, 75th percentile) or n (%)**		**Median (25th, 75th percentile) or n (%)**	**Median (25th, 75th percentile) or n (%)**	
Age (years)	686	21 (21, 22)	21 (21, 22)	21 (21, 22)	0.382	21 (21, 22)	21 (21, 22)	0.315	21 (21, 22)	21 (21, 22)	0.646
Race^b^ (Han)	676	576 (85.2)	522 (85.4)	54 (83.1)	0.611	469 (85.4)	107 (84.3)	0.737	529 (85.2)	47 (85.5)	0.957
Weight (kg)	681	63.10 (58.30, 70.35)	63.10 (58.20, 70.48)	62.90 (59.65, 69.95)	0.894	63.20 (58.30, 70.50)	62.70 (57.73, 69.78)	0.301	63.10 (58.18, 70.50)	62.90 (59.20, 69.20)	0.808
Height (cm)	681	173 (170, 176)	173 (170, 176)	174 (169, 177)	0.624	173 (170, 176)	173 (170, 176)	0.630	173 (170, 176)	174 (170, 178)	0.195
BMI (kg/m^2^)	681	21.28 (19.65, 23.09)	21.28 (19.70, 23.10)	21.26 (19.41, 22.88)	0.859	21.25 (19.76, 23.14)	21.31 (19.30, 22.76)	0.266	21.29 (19.63, 23.12)	21.23 (19.77, 22.60)	0.626
Body fat (%)	681	15.60 (13.20, 19.50)	15.70 (13.30, 19.50)	15.30 (13.00, 19.35)	0.831	15.70 (13.50, 19.70)	15.35 (12.60, 18.85)	0.204	15.70 (13.30, 19.53)	14.80 (12.80, 18.20)	0.214
**Sleep latency**^**c**^ **(minutes)**	686	30.00 (15.00, 50.00)	**30.00 (10.00, 47.25)**	**30.00 (20.00, 50.00)**	**0.021**	30.00 (10.00, 49.50)	30.00 (15.00, 50.00)	0.109	30.00 (15.00, 50.00)	30.00 (15.00, 50.00)	0.443
Sleep duration (hours)	686	7.00 (6.50, 8.00)	7.00 (6.50, 8.00)	7.00 (6.00, 8.00)	0.659	7.00 (6.50, 8.00)	7.00 (6.50, 8.00)	0.300	7.00 (6.50, 8.00)	7.00 (6.00, 8.00)	0.224
**Computer usage time (hours/day)**	686	4.00 (3.00, 6.00)	**4.00 (3.00, 6.00)**	**5.00 (3.00, 6.00)**	**0.011**	4.00 (3.00, 6.00)	4.00 (3.00, 6.00)	0.222	4.00 (3.00, 6.00)	4.00 (3.00, 5.50)	0.436
Time remaining seated (≥ 6 hours/day)	686	457 (66.6)	407 (65.6)	50 (75.8)	0.098	371 (66.7)	86 (66.2)	0.901	417 (66.1)	40 (72.7)	0.316
Physical activity (≥ 3 times/week)	682	237 (34.8)	218 (35.4)	19 (28.8)	0.284	195 (35.2)	42 (32.8)	0.609	217 (34.6)	20 (37.0)	0.713
Current tobacco smoking	685	153 (22.3)	136 (22.0)	17 (25.8)	0.483	127 (22.9)	26 (20.0)	0.477	143 (22.7)	10 (18.2)	0.441
Current alcohol drinking	686	511 (74.5)	462 (74.5)	49 (74.2)	0.961	414 (74.5)	97 (74.6)	0.971	471 (74.6)	40 (72.7)	0.755
Current tea consumption	686	155 (22.6)	145 (23.4)	10 (15.2)	0.128	130 (23.4)	25 (19.2)	0.308	146 (23.1)	9 (16.4)	0.249
Current coke drinking	686	431 (62.8)	383 (61.8)	48 (72.7)	0.080	341 (61.3)	90 (69.2)	0.093	391 (62.0)	40 (72.7)	0.111
Current coffee drinking	686	199 (29.0)	179 (28.9)	20 (30.3)	0.807	165 (29.7)	34 (26.2)	0.426	179 (28.4)	20 (36.4)	0.210
**Current milk tea drinking**	686	226 (32.9)	198 (31.9)	28 (42.4)	0.085	**172 (30.9)**	**54 (41.5)**	**0.021**	**201 (31.9)**	**25 (45.5)**	**0.040**
**Fried food consumption (≥1 time/week)**	686	303 (44.2)	**263 (42.4)**	**40 (60.6)**	**0.005**	**224 (40.3)**	**79 (60.8)**	**<** **0.001**	**270 (42.8)**	**33 (60.0)**	**0.014**
Baked food consumption (≥ 1 time/week)	686	125 (18.2)	109 (17.6)	16 (24.2)	0.183	99 (17.8)	26 (20.0)	0.560	115 (18.2)	10 (18.2)	0.994

Values were presented as median (25th, 75th percentile) or number (%). The results in bold indicate that there were significantly differences in the viable between different status of depression, anxiety, and stress symptoms (*p* < 0.05).

a Comparison between different status of depression, anxiety, and stress symptoms: continuous variable (Mann-Whitney *U*-test), categorical variables (χ^2^-test).

b Han people are an ethnic group native to East Asiathe, and they are world's largest ethnic group with more than 1.3 billion people.

^c^Time from going to bed to falling asleep.

**Table 2:** The distribution of demographic characteristics and lifestyle factors according to the status of depression, anxiety, and stress symptoms among 582 male college students at follow-up (year 2015).

**Variables**	**All subjects**		**Depression symptom**		***p*-values^a^**	**Anxiety symptom**		***p*-values^a^**	**Stress symptom**		***p*-values^a^**
	**(*****N*** = **582)**		**No (*n* = 543)**	**Yes (*n* = 39)**		**No (*n* = 508)**	**Yes (*n* = 74)**		**No (*n* = 543)**	**Yes (*n* = 39)**	
	**n**	**Median (25th, 75th percentile) or n (%)**	**Median (25th, 75th percentile) or n (%)**	**Median (25th, 75th percentile) or n (%)**		**Median (25th, 75th percentile) or n (%)**	**Median (25th, 75th percentile) or n (%)**		**Median (25th, 75th percentile) or n (%)**	**Median (25th, 75th percentile) or n (%)**	
**Age (years)**	582	22 (22, 23)	**22 (22, 23)**	**22 (21, 22)**	**0.002**	22 (22, 23)	22 (22, 23)	0.241	22 (22, 23)	22 (21, 23)	0.168
Race^b^ (Han)	582	486 (83.5)	452 (83.2)	34 (87.2)	0.522	422 (83.1)	64 (86.5)	0.460	450 (82.9)	36 (92.3)	0.125
Weight (kg)	581	65.10 (59.55, 72.20)	65.10 (59.58, 72.10)	65.60 (58.80, 73.10)	0.790	64.90 (59.40, 72.10)	66.15 (61.43, 72.50)	0.408	64.90 (59.38, 72.10)	66.10 (61.80, 73.10)	0.275
Height (cm)	582	173 (169, 176)	173 (169, 176)	175 (170, 178)	0.143	173 (169, 176)	173 (170, 178)	0.234	173 (169, 176)	175 (170, 179)	0.118
BMI (kg/m^2^)	581	21.76 (20.21, 23.73)	21.76 (20.21, 23.76)	21.86 (20.18, 23.31)	0.737	21.77 (20.21, 23.70)	21.58 (20.18, 23.84)	0.767	21.76 (20.17, 23.72)	21.75 (20.31, 23.77)	0.807
Body fat (%)	580	17.35 (14.03, 21.30)	17.20 (14.00, 21.05)	18.00 (14.90, 22.50)	0.466	17.10 (14.00, 21.30)	17.90 (14.05, 21.40)	0.748	17.20 (14.00, 21.25)	18.40 (14.40, 21.50)	0.357
**Sleep latency**^**c**^ **(minutes)**	582	25.00 (10.00, 40.00)	25.00 (10.00, 40.00)	20.00 (10.00, 40.00)	0.739	**20.00 (10.00, 30.00)**	**30.00 (10.00, 46.25)**	**0.046**	25.00 (10.00, 40.00)	30.00 (10.00, 40.00)	0.698
**Sleep duration (hours)**	581	7.00 (7.00, 8.00)	7.00 (7.00, 8.00)	7.50 (7.00, 8.00)	0.266	7.00 (7.00, 8.00)	7.50 (7.00, 8.00)	0.536	**7.00 (7.00, 8.00)**	**7.75 (7.00, 8.00)**	**0.035**
**Computer usage time (hours/day)**	582	6.00 (4.00, 8.00)	**6.00 (4.00, 8.00)**	**7.50 (5.00, 10.00)**	**0.001**	6.00 (4.00, 8.00)	6.00 (4.00, 10.00)	0.065	6.00 (4.00, 8.00)	6.00 (4.00, 10.00)	0.056
**Time remaining seated (≥6 hours/day)**	582	396 (68.0)	362 (66.7)	34 (87.2)	0.008	**338 (66.5)**	**58 (78.4)**	**0.041**	369 (68.0)	27 (69.2)	0.869
Physical activity (≥ 3 times/week)	582	168 (28.9)	156 (28.7)	12 (30.8)	0.786	148 (29.1)	20 (27.0)	0.709	156 (28.7)	12 (30.8)	0.786
Current tobacco smoking	582	131 (22.5)	121 (22.3)	10 (25.6)	0.628	115 (22.6)	16 (21.6)	0.845	118 (21.7)	13 (33.3)	0.094
Current alcohol drinking	581	435 (74.9)	409 (75.5)	26 (66.7)	0.221	383 (75.5)	52 (70.3)	0.329	407 (75.1)	28 (71.8)	0.647
Current tea consumption	582	134 (23.0)	127 (23.4)	7 (17.9)	0.436	122 (24.0)	12 (16.2)	0.136	128 (23.6)	6 (15.4)	0.241
Current coke drinking	582	347 (59.6)	320 (58.9)	27 (69.2)	0.205	302 (59.4)	45(60.8)	0.823	320 (58.9)	27 (69.2)	0.205
Current coffee drinking	582	146 (25.1)	133 (24.5)	13 (33.3)	0.219	126 (24.8)	20 (27.0)	0.680	137 (25.2)	9 (23.1)	0.764
Current milk tea drinking	582	156 (26.8)	145 (26.7)	11 (28.2)	0.838	140 (27.6)	16 (21.6)	0.281	148 (27.3)	8 (20.5)	0.358
**Fried food consumption (≥1 time/week)**	582	232 (39.9)	**207 (38.1)**	**25 (64.1)**	**0.001**	**188 (37.0)**	**44 (59.5)**	**<** **0.001**	**208 (38.3)**	**24 (61.5)**	**0.004**
Baked food consumption (≥ 1 time/week)	582	93 (16.0)	86 (15.8)	7 (17.9)	0.728	79 (15.6)	14 (18.9)	0.460	84 (15.5)	9 (23.1)	0.210

Values were presented as median (25th, 75th percentile) or number (%). The results in bold indicate that there were significantly differences in the viable between different status of depression, anxiety, and stress symptoms (*p* < 0.05).

^a^Comparison between different status of depression, anxiety, and stress symptoms: continuous variable (Mann-Whitney *U-*test), categorical variables (χ^2^-test).

^b^Han people are an ethnic group native to East Asiathe, and they are world's largest ethnic group with more than 1.3 billion people.

^c^Time from going to bed to falling asleep.

### Univariate analyses of the association of demographic and lifestyle factors with mental health problems

At baseline (Table 1), the results of univariate analyses showed that (i) subjects with depression symptoms had longer sleep latency [median (25th to 75th percentile) = 30.00 (20.00, 50.00) vs. 30.00 (10.00, 47.25) [min], *p* = 0.021], more computer usage time [5.00 (3.00, 6.00) vs. 4.00 (3.00, 6.00) [h/day], *p* = 0.011], and higher frequency of fried food consumption (percentage = 60.6% vs. 42.4%, *p* = 0.005) compared to subjects without depression symptoms, (ii) subjects with anxiety symptoms had higher frequency of current milk tea drinking (percentage = 41.5 vs. 30.9%, *p* = 0.021) and fried food consumption (percentage = 60.8 vs. 40.3%, *p* < 0.001) compared to subjects without anxiety symptoms, and (iii) subjects with stress symptoms had higher frequency of current milk tea drinking (frequency ≥ 1 time/week, percentage = 45.5 vs. 31.9%, *p* = 0.040) and fried food consumption (frequency ≥ 1 time/week, percentage = 60.0 vs. 42.8%, *p* = 0.014) compared to subjects without stress symptoms.

As shown in Table 2, the results of follow-up were similar to the baseline results. The data showed that (i) subjects with depression symptoms had lower age [22 (21, 22) vs. 22 (22, 23) [years], *p* = 0.002], higher computer use time [7.50 (5.00, 10.00) vs. 6.00 (4.00, 8.00) [h/day], *p* = 0.001], and higher frequency of fried food consumption (frequency ≥ 1 time/week, percentage = 64.1% vs. 38.1%, *p* = 0.001) compared to subjects without depression symptoms, (ii) subjects with anxiety symptoms had longer sleep latency [30.00 (10.00, 46.25) vs. 20.00 (10.00, 30.00) [min], *p* = 0.046], more time remaining seated (≥ 6 h/day, percentage = 78.4% vs. 66.5%, *p* = 0.041), and higher frequency of fried food consumption (frequency ≥ 1 time/week, percentage = 59.5% vs. 37.0%, *p* < 0.001) compared to subjects without anxiety symptoms, and (iii) subjects with stress symptoms had longer sleep duration [7.75 (7.00, 8.00) vs. 7.00 (7.00, 8.00) [h], *p* = 0.035] and higher frequency of fried food consumption (frequency ≥ 1 time/week, percentage = 61.5% vs. 38.3%, *p* = 0.004) compared to subjects without stress symptoms.

The above results collectively identified the following six factors, including age, computer usage time, fried food consumption, sleep latency, current milk tea drinking, and time remaining seated, as potential risk factors of mental health problems among college students.

### Multiple logistic regressions analyses of the association of demographic and lifestyle factors with mental health problems

Multiple logistic regressions were performed to further confirm the associations between the above-identified risk factors and the prevalence of depression, anxiety, and stress symptoms. As shown in Table 3, we found that (i) computer usage time was an independent lifestyle-related risk factor associated with the prevalence of depression symptom in both years and (ii) fried food consumption was an independent lifestyle-related risk factor associated with the prevalence of depression, anxiety and stress symptoms in 2 years. In detail, subjects with more computer usage time had higher risk of having depression symptoms compared to subjects with lower computer usage time at baseline (OR 2.18, 95% confidence interval[CI] = 1.29–3.68, *p* = 0.004) and follow-up (OR 1.97, 95% CI = 1.01–3.87, *p* = 0.048). Subjects with high frequency of fried food consumption had higher risk of having depression (OR 2.01, 95% CI = 1.19–3.39, *p* = 0.009 and OR 2.59, 95% CI = 1.30–5.13, *p* = 0.007), anxiety (OR 2.22, 95% CI = 1.49–3.30, *p* < 0.001 and OR 2.49, 95% CI = 1.50–4.12, *p* < 0.001), and stress (OR 1.85, 95% CI = 1.05–3.26, *p* = 0.035 and OR 2.72, 95% CI = 1.39–5.34, *p* = 0.004) symptoms compared to subjects with lower frequency of fried food consumption at baseline and follow-up, respectively.

**Table 3:** Association of the prevalence of depression, anxiety, and stress symptoms with lifestyle-related risk factors in 2 years.

**Risk factors**	**Year 2014** ^a^				**Year 2015** ^a^			
	**B (SE)**	**Wald**	**OR (95%CI)**	***p*-values**	**B (SE)**	**Wald**	**OR (95%CI)**	***p*-values**
Depression								
Age^b^	−0.17 (0.28)	0.36	0.85 (0.49,1.46)	0.550	−0.10 (0.43)	5.30	0.37 (0.16,0.86)	0.021
**Computer usage time** ^c^	**0.78 (0.27)**	**8.44**	**2.18 (1.29,3.68)**	**0.004**	**0.68 (0.34)**	**3.91**	**1.97 (1.01,3.87)**	**0.048**
**Fried food consumption**	**0.70 (0.27)**	**6.75**	**2.01 (1.19,3.39)**	**0.009**	**0.95 (0.35)**	**7.37**	**2.59 (1.30,5.13)**	**0.007**
Sleep latency^d^	0.41 (0.26)	2.42	1.51 (0.90,2.53)	0.120	−0.07 (0.34)	0.04	0.94 (0.48, 1.82)	0.849
Anxiety								
Current milk tea drinking	0.36 (0.20)	3.16	1.44 (0.96,2.15)	0.076	−0.39 (0.31)	1.62	0.68 (0.37,1.23)	0.203
**Fried food consumption**	**0.80 (0.20)**	**15.47**	**2.22 (1.49,3.30)**	**<** **0.001**	**0.91 (0.26)**	**12.47**	**2.49 (1.50,4.12)**	**<** **0.001**
Sleep latency^d^	0.23 (0.20)	1.36	1.26 (0.85,1.87)	0.244	**0.51 (0.26)**	**3.90**	**1.66 (1.00,2.76)**	**0.048**
Time remaining seated	−0.10 (0.21)	0.24	0.90 (0.60,1.37)	0.627	0.52 (0.88)	2.97	1.69 (0.93,3.06)	0.085
Stress								
Current milk tea drinking	0.50 (0.29)	2.97	1.64 (0.93,2.88)	0.085	−0.48 (0.41)	1.32	0.62 (0.28,1.40)	0.250
**Fried food consumption**	**0.61 (0.29)**	**4.45**	**1.85 (1.05,3.26)**	**0.035**	**1.00 (0.34)**	**8.48**	**2.72 (1.39,5.34)**	**0.004**
Sleep duration^e^	−0.24 (0.30)	0.64	0.79 (0.44,1.42)	0.786	**0.81 (0.35)**	**5.25**	**2.25 (1.12,4.49)**	**0.022**

The results in bold indicate that there were significantly differences in the viable between different status of depression, anxiety, and stress symptoms (*p* < 0.05). The risk factors in bold indicate that the results were in consistent in both 2 years.

a Logistic regression analyses were used to explore the associations between risk factors and the prevalence of depression, anxiety, and stress symptoms.

b Age was convert into dichotomous variable by its median value (median = 21 years).

c Computer usage time was convert into dichotomous variable by its median value (median = 4.00 h).

d Sleep latency was convert into dichotomous variable by its median value (median = 30.00 min).

e Sleep duration was convert into dichotomous variable by its median value (median = 7.00 h).

### Longitudinal analyses from baseline to follow-up

Based on the results of Table 3, computer usage time and fried food consumption are the only two variables associated with mental health outcomes in both 2 years. Thus, cross-lagged models were used to further explore the potential longitudinal associations of computer usage time and fried food consumption with prevalence of depression, anxiety, and stress symptoms. Overall, 552 subjects participated in both baseline and follow-up. The cross-lagged analysis (Figure 1) indicated that fried food consumption is a predictor of depression, anxiety, and stress status. In detail, fried food consumption in 2014 is positively correlated with depression (β = 0.129, *p* < 0.01), anxiety (β = 0.168, *p* < 0.001), and stress (β = 0.113, *p* < 0.01) status a year later. Results of Figure 2 indicated that computer usage time is a predictor of depression status but not anxiety and stress status. Data showed that computer usage time in 2014 is positively correlated with depression status (β = 0.106, *p* < 0.05) but not anxiety (β = 0.047, *p* > 0.05) and stress (β = 0.019, *p* > 0.05) status a year later. Collectively, these results provide additional evidence demonstrating that computer usage time and fried food consumption are two lifestyle-related risk factors linked to mental health problems over time, in our case, 1 year.

## Discussion

This study investigated the prevalence of mental health problems, including depression, anxiety, and stress symptoms, in 686 Chinese male college students longitudinally for 2 years and explored the lifestyle-related risk factor for mental health problems. Our findings indicated that age, sleep latency, sleep duration, computer usage time, milk tea drinking, and fried food consumption were potential risk factors for college students' mental health problems. Longitudinal analyses further revealed that computer usage time and fried food consumption at baseline is correlated with depression, anxiety, and stress status a year later. These results implied that reducing computer usage and fried food consumption might promote college students' mental health.

Univariate analyses (Tables 1, 2) identified six lifestyle-related risk factors for mental health problems, including age, computer usage, fried food consumption, sleep latency, current milk tea drinking, and time remaining seated. Physical activity was not associated with mental health problems in this study, while other researchers found that physical activity is an important protective factor in reducing the risk of developing depression and anxiety (24, 25). The reason of the inconsistent conclusion between our study and the previous studies might be as following. First, physical activity level was measure by a question assessing physical activity frequency in the present study. While, the type of sport and the duration of each exercise would also affect physical activity level. For example, results from Grasdalsmoen et al. showed that physical exercise duration and intensity were both associated with mental health problems (26). Another reason might be the interactions effects of other social factors which we did not take into consideration in present study. For example, Vankim et al. reported that socializing might partially mediate the relationship between physical activity and mental health problems among college students (27). However, our data showed that more time remaining seated correlated with an increased risk of having anxiety symptoms, which is consistent with previous studies demonstrating a consistent association between sedentary behavior and poor mental health (28). Univariate analyses also revealed that milk tea drinking was potential lifestyle-related risk factor for college students' poor mental health. However, when adjusted for other confounders, no significant associations between milk tea drinking and mental health problems were detected. Although certain ingredients in tea, such as tea amino acid and L-theanine (L-THE), were reported to be associated with several mental health benefits (12), the underlying molecular mechanisms remained unclear. The associations of tea or tea products with mental health benefits need to be further studied and verified.

Results of multiple logistic regressions analysis further confirm a positive relationship between fried food consumption and computer usage time with mental health problems in college students at baseline or during follow-up. In order to explore whether lifestyle-related risk factors at baseline predict the prevalence of mental health a year later, the cross-lagged analysis were conducted. Our data showed that computer usage time and fried food consumption at baseline is correlated with depression, anxiety, and stress status a year later. These results indicate that reducing the use of computers and the frequency of eating fried food is beneficial to college students' mental health. For fried food consumption, the research by Yoshikawa et al. demonstrated that the frequency of fried food consumption was associated with lower resilience to depression, which further confirmed our results (29). Fried foods were usually cooked with large amounts of vegetable oil which have been demonstrated to elevate the risk of mental disorders, perhaps due to the imbalance in the intake of long-chain n-6 polyunsaturated fatty acid (LC n-6 PUFA) and long-chain n-3 polyunsaturated fatty acid (LC n-3 PUFA) (30). Based on our data, the proportion of college students who eat barbecue and drink milk tea is very high; thus, dietary behaviors are expected to become an important means to promote mental health in college students. Another factor worthy of attention was computer usage. Previous studies revealed that using the computer without breaks was a risk factor for several mental health outcomes (31). Consistent with previous studies, the present study revealed that subjects with depressive symptoms scored higher on computer usage compared to those who did not have depression symptoms at both baseline and follow-up. Previous studies linked heavy computer use to disturbed sleep (32), while poor sleep quality is important factor which affects mental health (6, 33). Researchers also found that sedentary behavior is associated with poor mental health (8), while computer use is usually associated with increased sedentary time. The above evidence raise a hypothesis that sleep and sedentary behavior might mediated the correlation between computer use and mental health. A recent study reported that the association between screen time (such as web surfing and computer gaming) and depressive symptoms is partially or fully mediated by sleep (34), which might support our hypothesis. Furthermore, since the participants' computer usage time increased notably during 1 year, paying attention to its adverse effects on mental health was greatly important.

Although prospective studies regarding lifestyle-related risk factors and mental health problems are limited, the findings of the following studies are still of great value. For example, a prospective study conducted with Swedish health care workers and social insurance officers concluded that physical activity participation appeared to lower the risk of developing mental health problems 2 years later (35). The results of another longitudinal study that aimed to investigate the temporal relationship between lifestyle and mental health among 564 midlife women demonstrated that BMI and current non-caffeine drinking were positively related to mental well-being while smoking had adverse effects (36). A study conducted in Japanese high school students showed that sports activities, time per day of studying outside school, gender, sleep quality, poor appetite, and watching television were either protective or risk factors for poor mental health (37). The results of a recent longitudinal study also conducted with university students, similar to our study, found that university socio-cultural environment and students' lifestyle factors, such as being female, financial condition, screen time, and sleep duration, had adverse effects on psychological health conditions of the college students (38). Our study did not found significant associations of BMI, smoking, and coffee drinking etc. with mental health outcomes, which was inconsistent with the results of several prospective studies mentioned above. The reasons might be as follows: 1) First, lifestyle behaviors and prevalence of mental health problems vary across different races, countries, and cultures (5). The different conclusion might arise from population bias; 2) Second, lifestyle behaviors and mental health status were also different between males and females (14, 15). As subjects in our study are all young males, gender difference is an important aspect to be aware of; 3) Third, the characteristics of lifestyle behaviors among college students are distinctive. For example, the proportion of students who use computers for a long time is relatively higher than their peers, while hyperadiposity is not common among Chinese people, especially in college students. It might explain why the present study detected an association of mental health problems with computer usage time, but not with BMI among college students.

Although our study was established in 2013, another issue worth discussing is the impact of COVID-19 pandemic on college student mental health. A recent study reported that about two-fifths college students in China experienced anxiety symptoms during the COVID-19 epidemic (39). Researchers also found that lifestyle-related factors such as BMI, physical activity, eating habits, and learning environment were markedly changed during the COVID-19 pandemic, which might associated increased prevalence of mental health problems and suicide rate among college students (40–42). Thus, focusing on those lifestyle-related factors that might be affected by the COVID-19 pandemic and promoting risk perceptions and preventive practices are of great importance in future studies (43).

### Limitations

The present study has several limitations. First, all subjects were from the MARHCS cohort study; thus, they were all male college students. A potential gender bias regarding the prevalence of mental health problems and lifestyle characteristics should not be ignored (44). Generalizability of our results should be aware of, and the conclusion of our study needs further verification in other studies. Second, since the DASS-21 does not have clinically proven validity, caution should be exercised in generalizing our conclusions to a clinical context (45). Third, we used self-reported data on lifestyle factors such as sleep quality, physical activity, and dietary habit, which might introduce potential error or bias into findings of the present study. Fourth, unlike cross-sectional studies, we used longitudinal analysis methods to examine the potential causal relationship. This study design may help to analyze the temporal relationship between lifestyles and mental health problems. However, solid cause-and-effect conclusions still need validation in large sample multi-center randomized controlled studies. Fifth, using propaganda poster in university campuses and online recruitment, our study was open to all the male college students in Chongqing who met our inclusion criteria. Since we did not use a peer-to-peer questionnaire method, the number of the subjects who received our recruitment information is unclear. Thus, response rate of the present study was not provided. Sixth, remuneration might bring bias to the representativeness of sample and also affect response rate. Finally, this study did not assess social-cultural factors such as social desirability which might also affect an individual's mental health conditions. For example, Fastame et al. reported that social desirability would contaminate psychological well-being in late adulthood (46). The impact of social-cultural factors on mental health deserves special attention in future research.

## Conclusion

Overall, in this longitudinal observational study of 686 male college students from China, we identified six lifestyle-related risk factors for mental health problems. Further longitudinal analyses demonstrated that computer usage time and fried food consumption at baseline predict the prevalence of mental health a year later. To the best of our knowledge, this was the first cohort study to report the longitudinal association between lifestyle-related risk factors and mental health problems in Chinese college students. The results of our study might provide further preventive strategies for mental health problems.

## Data availability statement

The raw data supporting the conclusions of this article will be made available by the authors, without undue reservation.

## Ethics statement

The studies involving human participants were reviewed and approved by the Institutional Review Board of the Third Military Medical University, Chongqing, China. The patients/participants provided their written informed consent to participate in this study.

## Author contributions

JC and LA: study design, manuscript review, and project supervision. B-WY and PZ: manuscript draft and edit. QC and HY: data analyses. LS, XL, N-YZ, L-HW, L-PH, J-YL, and H-FY: data collection. Special thanks to Prof. QC and HY for their advice and selfless help during revision. All authors contributed to the article and approved the submitted version.

## Funding

This study was supported by the National Natural Science Foundation of China (No. 82003429).

## Conflict of interest

The authors declare that the research was conducted in the absence of any commercial or financial relationships that could be construed as a potential conflict of interest.

## Publisher's note

All claims expressed in this article are solely those of the authors and do not necessarily represent those of their affiliated organizations, or those of the publisher, the editors and the reviewers. Any product that may be evaluated in this article, or claim that may be made by its manufacturer, is not guaranteed or endorsed by the publisher.
